# Gender and growth variation in palatal bone thickness and density for mini-implant placement

**DOI:** 10.1186/s40510-018-0241-1

**Published:** 2018-11-05

**Authors:** Sumit Yadav, Emily Sachs, Meenakshi Vishwanath, Kristen Knecht, Madhur Upadhyay, Ravindra Nanda, Aditya Tadinada

**Affiliations:** 1 Division of Orthodontics, University of Connecticut Health Center, Farmington, USA; 2 School of Dental Medicine, University of Connecticut Health Center, 263 Farmington Avenue, L7062A MC1725, Farmington, CT 06030 USA; 3 Division of Orthodontics, University of Nebraska Medical Center, Farmington, USA; 4 Private Practice, Houston, Texas USA; 5 Division of Oral Health and Diagnostic Sciences, University of Connecticut Health Center, Farmington, USA

## Abstract

**Background:**

The objective was to compare the palatal bone thickness (PBT) and palatal bone density (PBD) in the anterior, middle, and posterior part of the palate in males and females.

**Methods:**

This retrospective study reviewed cone beam computed tomography scans of 359 patients. The scans were divided into 99 growing males, 105 growing females, 74 non-growing males, and 81 non-growing females. The measurements of PBT and PBD were made in between the canine and first premolar, the first premolar and second premolar, the second premolar and first molar, and the first molar and second molar. The measurements were made in the center of the palate and 4 mm away from the center. ANOVA was used to analyze the PBT and PBD in different areas between four different groups.

**Results:**

The PBT was lower (*P* <  0.0001) as we moved from the anterior to the posterior palate. The PBT was more (*P* <  0.001) in the center of the palate than 4 mm away from the center, except in between the canine and first premolar. The growing male and non-growing male had higher (*P* <  0.0001) PBT than the growing female and non-growing female in between the canine and first premolar and the first premolar and second premolar both in the center and 4 mm away from it. The PBD was higher (*P* <  0.05) in between the canine and first premolar area at the center of the palate and between the second premolar and first molar 4 mm away from the center in all the experimental groups.

**Conclusions:**

There exists a definite gender and growth variation in the PBT and PBD in different parts of the palate. Palatal bone thickness between the males and females revealed that the males had significantly higher PBT than the females.

## Background

In the recent years, palatal mini-implants have gained popularity and have been widely used for molar intrusion, molar protraction, segment protraction, and anterior tooth retraction [1–7]. The palatal mini-implants are usually preferred, because the site of mini-implant placement is easily accessible, has less soft tissue irritation, does not interfere with the desired orthodontic tooth movement, and has good quality and quantity of bone [8, 9]. The palatal mini-implants are commonly inserted in the anterior region of the palate, mid-palatal area, and the posterior region of the palate [9, 10]. The success of the mini-implants usually depends on the bone quantity (bone volume/amount of bone present) and bone quality (bone density). Bone quality and quantity can be influenced by many factors, including heredity, race, environment, nutrition, and lifestyle [11–13].

The success of mini-implants usually depends on the degree to which it integrates (mechanically and biologically) with the host bone. The palatal bone thickness and palatal bone density vary at different mini-implant insertion sites in the palate, and the appropriate knowledge of the amount of bone available (bone thickness/bone volume) and amount of bone mineralization (bone mineral density) should guide orthodontic clinicians to make educated decisions in selecting the mini-implant placement site. The mineral content of the bone matrix and heterogeneity of mineralization are important factors to assess the quality of bone. Palatal mini-implants have shown excellent stability. Karagkiolidou et al. showed that approximately 98% of mini-implants are stable, when they are inserted in the anterior region of the palate [14]. Manni et al. showed that the failure risk of mini-implants is significantly more in females when compared to males [15].

Three-dimensional imaging using cone beam computed tomography provides a method to characterize the bone quantity and bone mineral density (bone quality) [16, 17]. Studies have shown that cone beam computed tomography (CBCT) can be used for assessing the bone quantity and quality (bone mineral density) of the maxillary and mandibular bones [17, 18]. Bone mineral density is usually assessed in 3D imaging by evaluating the gray levels of the CBCT images [19–22]. It has been well documented that there exist a gender variation between the bone quantity and bone quality [19, 23]. Moreover, it has been shown that an adult (non-growing) has more bone mineral density than growing people [23].

Studies have assessed either the bone quality or the bone quantity individually; to our knowledge, our study is the first study to assess the bone quality and bone quantity in the same patients [1, 4, 24, 25]. Our objective is to measure the palatal bone thickness and palatal bone density at different sites in the palate (in the center of palate and 4 mm away from the center) in a population of growing and non-growing Caucasian subjects, who previously received CBCT scans for orthodontic treatment. Additionally, our aim is to compare and contrast the palatal bone thickness and palatal bone density between males (growing males vs. non-growing males) and females (growing females vs. non-growing females). Our null hypothesis is that the palatal bone thickness and palatal bone density at different sites is not different between males and females.

## Methods

An institutional review board exemption was obtained for evaluating CBCT volumes archived in the Department of Oral and Maxillofacial Radiology. This retrospective study reviewed 359 CBCT scans of patients who were referred for orthodontic treatment. All CBCT scans were acquired using the iCAT Next Generation (Imaging Sciences International, Hatfield, Pa) CBCT unit. A standardized protocol of the iCAT for the extended (17 × 23 cm) field of view (FOV) with 0.3-mm slice thickness and 26.9-s acquisition time was used. All scans were saved in the DICOM-3 format and were evaluated using a third party CBCT reconstruction software InVivo5.0 (Anatomage, San Jose, CA). On the basis of age, the scans were divided into four groups: group 1, growing male; group 2, growing female; group 3, non-growing male; and group 4, non-growing female. The exclusion criteria were (1) cases with congenitally missing teeth; (2) CBCT scans showing supernumerary teeth, enlarged/cystic follicle, or any other pathology; (3) CBCT scans showing no systemic disease affecting the bone of the patients; and (4) CBCT scans showing impacted teeth in the area of the measurement. These exclusion criteria were set, as these might have affected the palatal one thickness (PBT) and the palatal bone density (PBD).

**Fig. 1:**
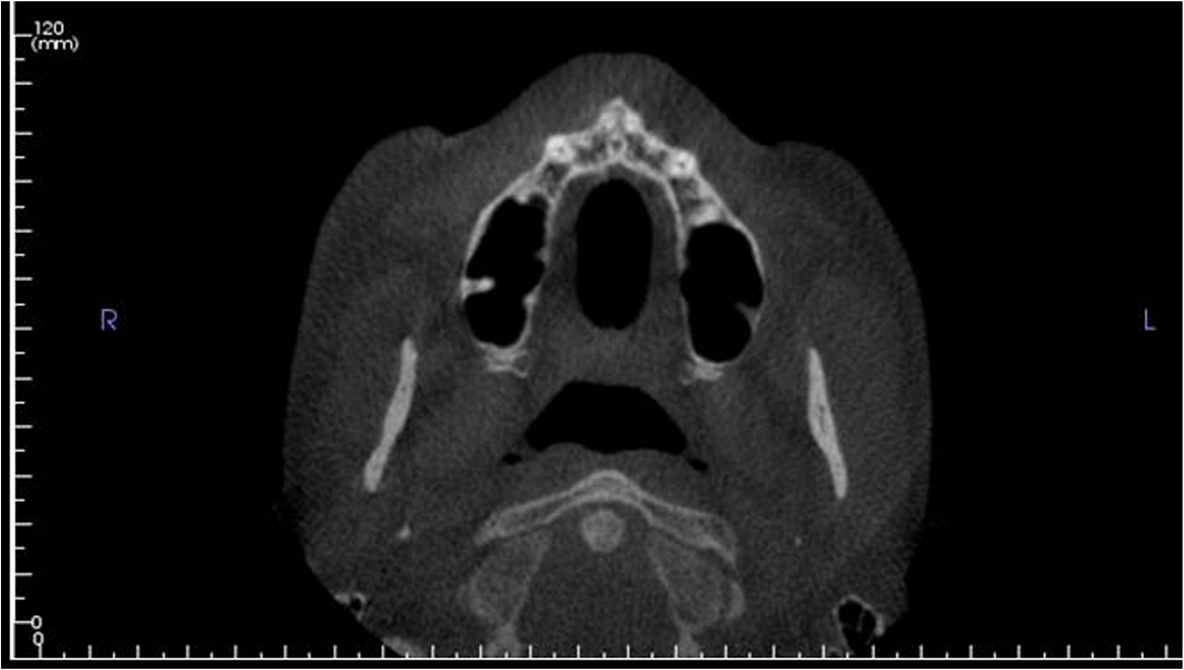
Axial section depicting the localization at the level of the incisive foramen

**Fig. 2:**
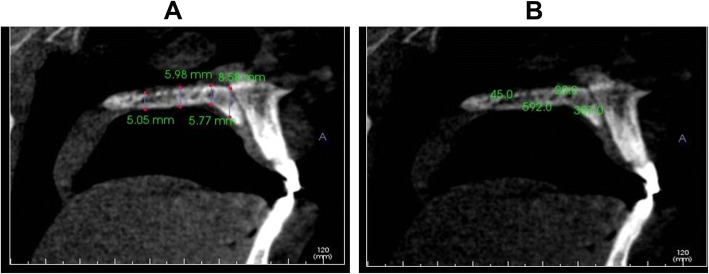
**a** Sagittal section showing the measurement of palatal bone thickness (PBT). **b** Sagittal section showing the measurement of palatal bone density (PBD)

All CBCT volumes were imported into Invivo5 (Ver. 5.3) (Anatomage Inc., CA) software, and a single examiner reviewed all the scans independently. The investigators reviewed the images on a split screen dual display monitor (HP Compaq LA2205wg) under standardized conditions of ambient light and sound. The investigators had the full capability to evaluate the volumes in all the three orthogonal planes and manipulate contrast and histogram. Once the scans were imported into the reconstruction program, all scans were aligned parallel to Frankfort’s horizontal plane. The scans were then aligned in the field of view on axial sections to be in the center of the palate using the incisive foramen as a standardized landmark (Fig. 1). Once the center was established on all the three planes (axial, sagittal, and coronal) using the toggled cross hairs in the program, PBT was measured. The measurements of PBT were made in between the canine and first premolar, between the first premolar and second premolar, between the second premolar and first molar, and between the first molar and second molar (Fig. 2a). The measurements were made in the center of the palate and 4 mm sequentially away from the center of the palate (Fig. 2a).

The measurements of PBD were made in between the canine and first premolar, between the first premolar and second premolar, between the second premolar and first molar, and between the first molar and second molar. Using the Hounsfield unit (HU) equivalent pixel intensity value scale in the software program, density values were recorded in the center of the palate (Fig. 2b). To test the intraexaminer reliability, 20 randomly selected scans were measured 4 weeks later by the same person both for PBT and PBD.

### Statistical approach

Descriptive statistics were used to summarize the outcomes (palatal bone thickness and palatal bone density). Mean, standard deviation, and percentile distributions were computed for palatal bone thickness and palatal bone density. Power analysis showed that a sample size of at least 30 subjects per group would give an 80% probability of detecting a real difference between the groups at a statistically significant level of 5%. Inter-examiner reliability was computed by Cronbach alpha values. A one-sample Kolmogorov–Smirnov test was used to examine normality of distribution of the outcomes measured. The outcomes were distributed normally, and one-way ANOVA was used to determine the significance between the different sites measured in different experimental groups. Tukey’s test was used for multiple comparisons between the groups. Pearson correlation coefficient (*r*) was used to measure the correlation between the PBT and PBD in the center of the palate in different experimental groups. Kappa statistics were done to measure the intraexaminer reliability. All statistical tests were two sided, and a *P* value of < 0.05 was deemed to be statistically significant. The size of the total measurement error (ME) was calculated with the following formula:$\mathrm{ME}=\sqrt{\Big(\Sigma}{d}^2\Big)/2n$ with *d* the difference between the two measurements and *n* the number of double measurements. The overall measurement error of the various measurements was not greater than 0.3 mm. Statistical analyses were computed using Graph Pad software (LaJolla, CA, USA).

## Results

A total of 359 patients were included in the study. This included 99 growing males (12 years and 5 months ± 3 years and 2 months), 74 non-growing males (27 years and 1 month ± 3 years and 9 months), 105 growing females (mean age, 13 years and 4 months ± 2 years and 1 month), and 81 non-growing females (mean age, 31 years and 3 months ± 4 years and 7 months). Cohen’s kappa was 0.87 for the intraexaminer reliability. Distribution of PBT at different sites is summarized in Tables 1, 2, 3, and 4. PBT was significantly lower (*P* <  0.0001) as we moved from the anterior palate to the posterior palate in all the groups (Tables 1, 2, 3, and 4). The PBT was significantly (*P* <  0.001) more in the center of the palate than 4 mm away from the center, except in between the canine and first premolar (Tables 1, 2, 3, and 4), where the PBT was significantly lower (*P* <  0.05) in the center than 4 mm away from it (growing male, 8.17 ± 2.38 < 11.73 ± 3.16 mm; growing female, 6.36 ± 2.17 < 9.74 ± 2.81 mm; non-growing male, 8.29 ± 2.88 < 12.63 ± 3.38 mm; non-growing female, 6.79 ± 2.64 < 9.57 ± 3.01 mm). The sex comparison showed significantly higher (*P* <  0.001) PBT in the male. The growing male had significantly higher (*P* <  0.0001) bone thickness than the growing female in between the canine and first premolar and between the first premolar and second premolar both in the center of the palate and 4 mm away from the palate (Tables 1 and 2). Similarly, non-growing male had significantly greater (*P* <  0.001) PBT than non-growing female in between the canine and first premolar and the first premolar and second premolar both in the center of the palate and 4 mm away from the palate (Tables 3 and 4). Further, we were not able to differentiate (*P* > 0.05) between the PBT between growing male and non-growing male at all the sites of measurements (Tables 1 and 3). Similarly, we were not able to differentiate (*P* > 0.05) between growing females and non-growing females (Tables 2 and 4).

**Table 1:** Descriptive statistics of palatal bone thickness in growing male

	Canine and first PM	Canine and first PM (4 mm)	First PM and second PM	First PM and second PM (4 mm)	Second PM and first M	Second PM and first M (4 mm)	First M and second M	First M and second M (4 mm)
Number of values	99	99	99	99	99	99	99	99
Minimum	2.89	5.15	1.51	1.27	0.92	1.04	0.97	0.65
25% Percentile	6.57	9.5	5.39	4.01	3.72	2.47	3.44	1.44
Median	8.24	11.4	6.45	5.37	5.15	3.43	4.43	2.26
75% Percentile	10.04	14.13	7.62	7.15	6.57	4.81	5.82	3.72
Maximum	13.91	19.06	11.92	10.99	11.09	9.3	11.34	7.66
Mean	8.17	11.73	6.465	5.582	5.269	3.709	4.727	2.716
Std. deviation	2.38	3.169	2.041	2.148	2	1.622	2.025	1.549
Std. error of mean	0.2392	0.3185	0.2051	0.2159	0.201	0.163	0.2035	0.1557
Lower 95% CI	7.696	11.09	6.058	5.154	4.87	3.385	4.323	2.407
Upper 95% CI	8.645	12.36	6.872	6.011	5.667	4.032	5.131	3.025

*PM* premolar, *M* molar

**Table 2:** Palatal bone thickness in growing female

	Canine and first PM	Canine and first PM (4 mm)	First PM and second PM	First PM and second PM (4 mm)	Second PM and first M	Second PM and first M (4 mm)	First M and second M	First M and second M (4 mm)
Number of values	105	105	105	105	105	105	105	105
Minimum	1.29	3.44	1.98	0.84	1.34	0.57	0.9	0.71
25% Percentile	4.805	7.695	4.32	3.26	3.81	2.295	3.05	1.475
Median	6.36	9.73	5.2	4.5	4.57	2.96	3.94	1.89
75% Percentile	7.91	11.81	6.775	5.92	6.055	4.115	5.27	2.865
Maximum	11.97	16.28	10.23	12.22	9.02	7.24	9.62	6.04
Mean	6.36	9.746	5.47	4.694	4.846	3.215	4.369	2.273
Std. deviation	2.177	2.819	1.838	1.98	1.637	1.397	1.84	1.18
Std. error of mean	0.2124	0.2751	0.1794	0.1932	0.1598	0.1363	0.1796	0.1151
Lower 95% CI	5.939	9.2	5.114	4.311	4.529	2.945	4.013	2.044
Upper 95% CI	6.782	10.29	5.826	5.077	5.163	3.486	4.725	2.501

*PM* premolar, *M* molar

**Table 3:** Descriptive statistics of palatal bone thickness in non-growing male

	Canine and first PM	Canine and first PM (4 mm)	First PM and second PM	First PM and second PM (4 mm)	Second PM and first M	Second PM and first M (4 mm)	First M and second M	First M and second M (4 mm)
Number of values	74	74	74	74	74	74	74	74
Minimum	1.38	4.36	1.41	1.46	1.07	1.13	0.8	0.77
25% Percentile	5.953	10.49	5.82	4.258	3.728	2.493	2.583	1.285
Median	8.21	13.14	6.965	5.845	5.265	3.645	3.995	1.94
75% Percentile	10.22	14.58	8.229	7.413	6.485	4.783	5.665	2.948
Maximum	14.71	20.36	13.1	11.26	11.21	8.86	9.76	5.66
Mean	8.297	12.63	7.032	5.991	5.273	3.729	4.156	2.24
Std. deviation	2.888	3.385	2.158	2.291	2.167	1.559	2.06	1.139
Std. error of mean	0.3357	0.3935	0.2509	0.2663	0.2519	0.1812	0.2395	0.1324
Lower 95% CI	7.628	11.84	6.532	5.46	4.771	3.368	3.679	1.976
Upper 95% CI	8.966	13.41	7.532	6.522	5.775	4.091	4.633	2.503

*PM* premolar, *M* molar

**Table 4:** Palatal bone thickness in non-growing female

	Canine and first PM	Canine and first PM (4 mm)	First PM and second PM	First PM and second PM (4 mm)	Second PM and first M	Second PM and first M (4 mm)	First M and second M	First M and second M (4 mm)
Number of values	81	81	81	81	81	81	81	81
Minimum	1.95	0.88	1.91	0.94	1.01	0.84	0.99	0.69
25% Percentile	4.565	7.65	4.165	2.935	3.485	2.07	2.88	1.43
Median	6.5	9.47	5.19	4.2	4.59	2.8	4.09	2.05
75% Percentile	8.72	11.37	6.265	5.595	5.695	3.81	5.51	2.94
Maximum	14.69	17.04	10.79	9.69	9.99	6.56	9.44	5.77
Mean	6.79	9.573	5.352	4.336	4.789	2.993	4.317	2.273
Std. deviation	2.64	3.019	1.804	1.891	1.901	1.281	1.994	1.094
Std. error of mean	0.2934	0.3355	0.2004	0.2101	0.2112	0.1424	0.2216	0.1215
Lower 95% CI	6.206	8.906	4.953	3.918	4.368	2.71	3.876	2.031
Upper 95% CI	7.373	10.24	5.751	4.754	5.209	3.277	4.758	2.515

*PM* premolar, *M* molar

**Fig. 3:**
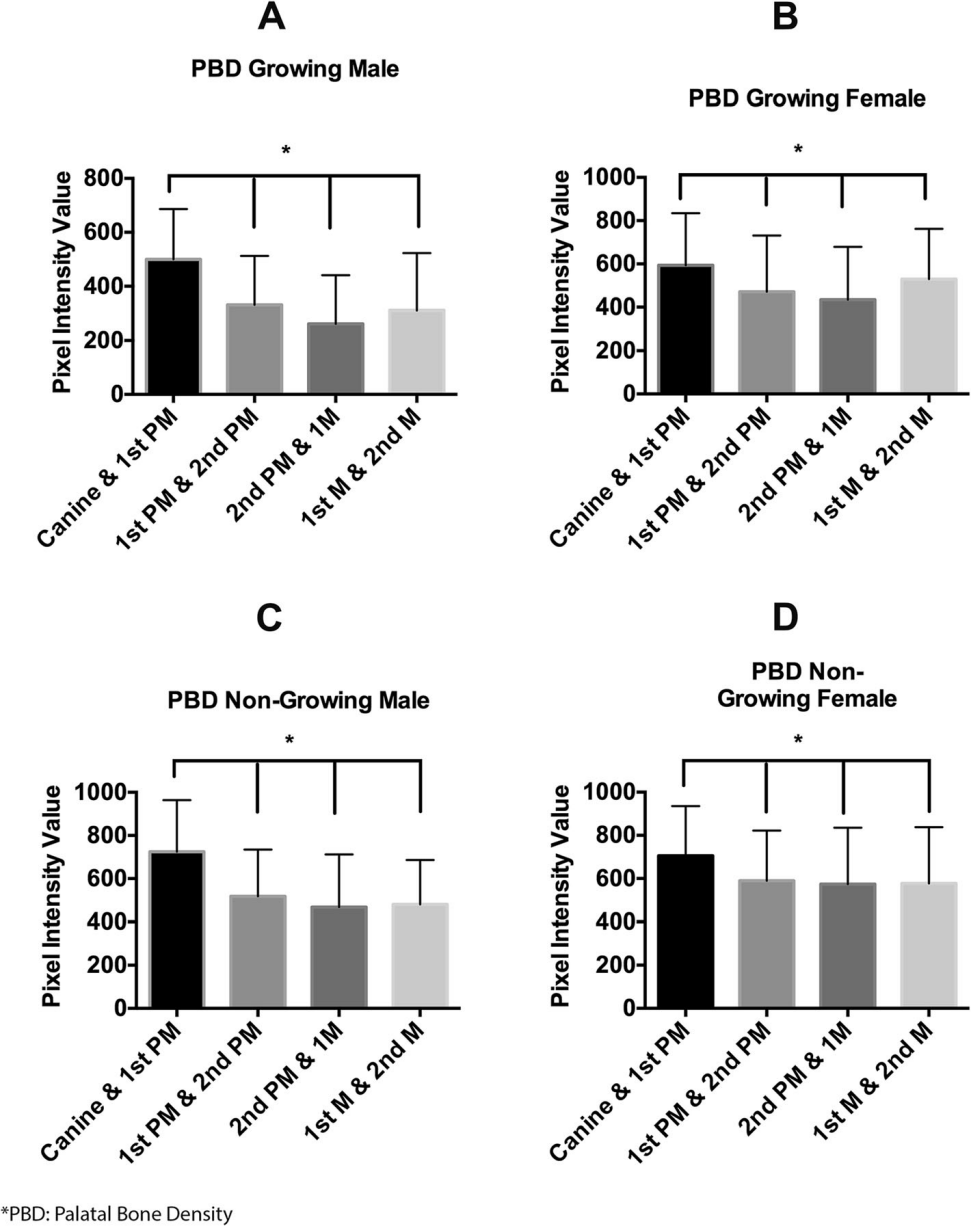
Bar graph showing the palatal bone density among different groups in the center of palate. **a** Palatal bone density in growing male at four different points measured. **b** Palatal bone density in growing female at four different points measured. **c** Palatal bone density in non-growing male at four different points measured. **d** Palatal bone density in non-growing female at four different points measured. Asterisk depicts that palatal bone density is significantly more in between the canine and first premolar when compared to other three sites measured

**Fig. 4:**
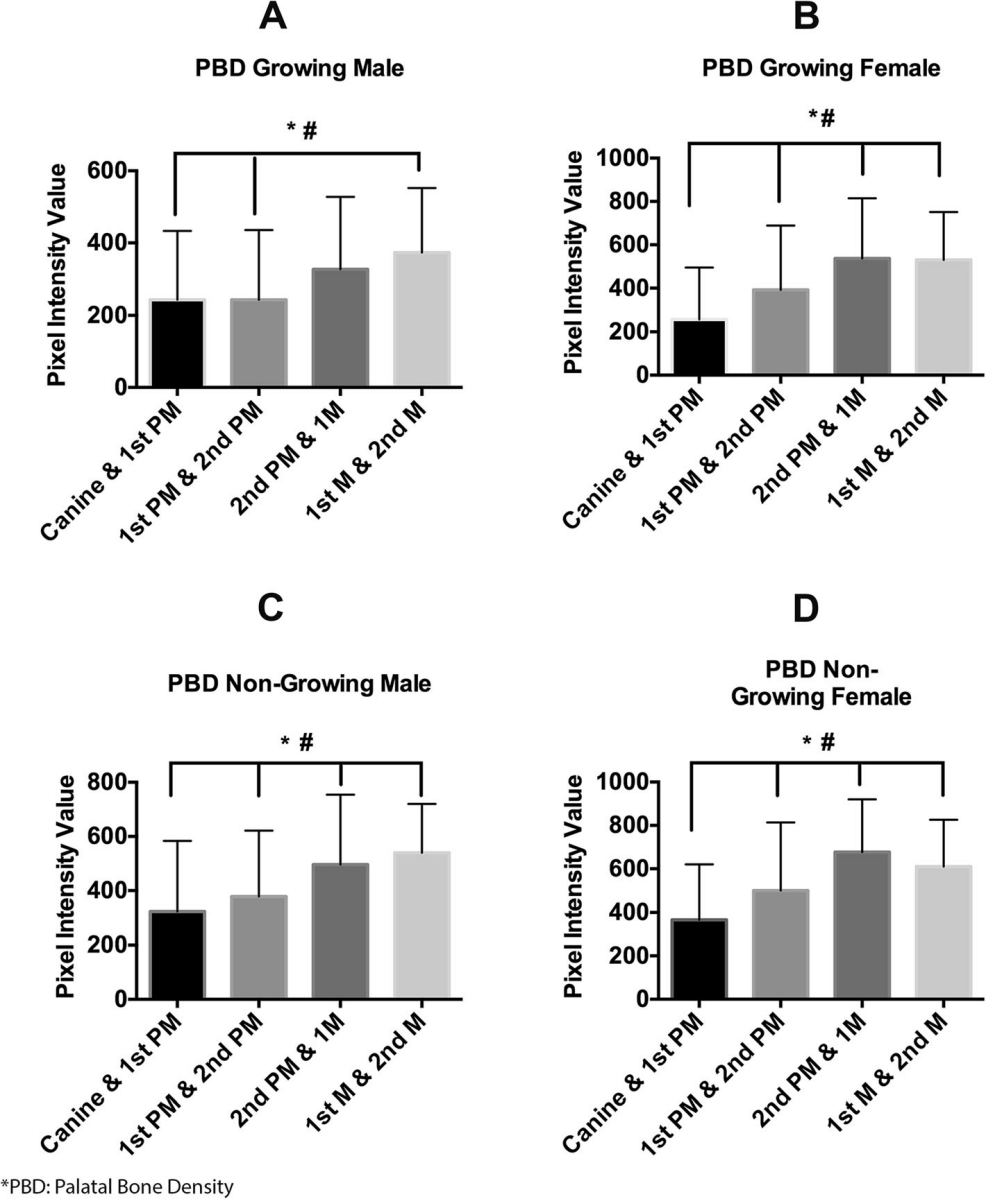
Bar graph showing the palatal bone density among different groups at 4 mm from the center of palate. **a** Palatal bone density in growing male at four different points measured. **b** Palatal bone density in growing female at four different points measured. **c** Palatal bone density in non-growing male at four different points measured. **d** Palatal bone density in non-growing female at four different points measured. Asterisk depicts that palatal bone density is significantly more in between the second premolar and first when compared to other canine and first premolar and between first premolar and second premolar. Number sign depicts that palatal bone density is significantly more in between the first molar and second molar and first when compared to other canine and first premolar and between first premolar and second premolar

The PBD was significantly higher (*P* <  0.005) in the canine and first premolar area in the center of the palate (Fig. 3), whereas in between the second premolar and first molar and between the first molar and second molar, it was significantly higher (*P* <  0.0001) 4 mm away from the center of the palate across all the experimental groups (Fig. 4). Growing female had a significantly higher (*P* < 0.05) PBD than growing male in the center of the palate and 4 mm away from the center, in all the areas except between the canine and first premolar, where we were not able to differentiate between growing female and growing male (Fig. 3). Similarly, non-growing female had a significantly higher bone density than growing male both in the center of the palate and 4 mm away from the center at all the sites measured, except between the canine and first premolar area (Fig. 4). As we moved laterally (4 mm away from the center), the palatal bone density increased significantly (*P* < 0.001) in between the second premolar and first molar and the first molar and second molar in all the groups (Fig. 4).

The correlation between PBT and PBD at the center of the palate between the canine and first premolar, the first premolar and second premolar, the second premolar and first molar, and the first molar and second premolar was significant (*P* < 0.05) (Table 5) and was negatively correlated in all the four experimental groups except in non-growing males in the canine and first premolar area (*P* = 0.9844) and growing males in the first premolar and second premolar area (*P* = 0.5363) (Table 5).

**Table 5:** Correlation between palatal bone thickness and palatal bone density in different groups

Experimental groups	Number of values (XY pairs)	Correlation coefficient	95% confidence interval	*P* value	Significant
Canine and first premolar					
Growing male	99	− 0.2582	“− 0.4335 to − 0.06405”	0.0099	Yes
Growing female	105	− 0.3799	“− 0.5327 to − 0.2030”	< 0.0001	Yes
Non-growing male	74	0.00231	“− 0.2263 to 0.2307”	0.9844	No
Non-growing female	81	− 0.3	“− 0.4865 to − 0.08735”	0.0065	Yes
First premolar and second premolar					
Growing male	99	0.06289	“− 0.1362 to 0.2571”	0.5363	No
Growing female	105	− 0.3251	“− 0.4864 to − 0.1423”	< 0.0001	Yes
Non-growing male	74	− 0.2713	“− 0.4706 to − 0.04561”	0.0194	Yes
Non-growing female	81	− 0.352	“− 0.5297 to − 0.1448”	0.0013	Yes
First premolar and second premolar					
Growing male	99	− 0.2687	“− 0.4426 to − 0.07530”	0.0072	Yes
Growing female	105	− 0.4163	“− 0.5630 to − 0.2441”	< 0.0001	Yes
Non-growing male	74	− 0.4364	“− 0.6046 to − 0.2309”	0.0001	Yes
Non-growing female	81	− 0.4959	“− 0.6445 to − 0.3112”	< 0.0001	Yes
First molar and second molar					
Growing male	99	− 0.3938	“− 0.5486 to − 0.2129”	< 0.0001	Yes
Growing female	105	− 0.4315	“− 0.5756 to − 0.2615”	< 0.0001	Yes
Non-growing male	74	− 0.3707	“− 0.5524 to − 0.1553”	0.0012	Yes
Non-growing female	81	− 0.1825	“− 0.3855 to 0.03738”	0.103	No

## Discussion

Skeletal anchorage has enabled clinicians to perform orthodontic tooth movement in all the three planes of space efficiently. The palatal bone has gained enormous popularity for the placement of skeletal anchorage devices for the treatment of complex malocclusions. The anatomic location of the palatal bone offers adequate keratinized soft tissue and little to no root injury during the mini-implant placement and does not interfere with majority of orthodontic tooth movement. The present study was undertaken to simultaneously evaluate both the palatal bone thickness and palatal bone density at different points in the palate using 3D CBCT scans. Previous studies have individually investigated either the palatal bone thickness or the palatal bone density, but none of them have studied them in similar patients and none of the previous studies have evaluated and correlated both variables (palatal bone thickness and palatal bone density) in different regions of the anterior, middle, and posterior palate [1, 20, 24, 25]. The other important aspect of our study was our large sample size, which afforded us the ability to segment the CBCT scans into four groups and yet allow each segment to be large; thus, supporting high-quality sampling that was more representative of the population and limiting the influence of outliers or extreme observations. Further, we did measure the palatal bone density at different sites, as there exist a regional variation in the bone density in the alveolar bone.

Our null hypothesis was rejected, as there was a statistically significant variation in the PBT between the anterior, middle, and posterior part of the palate (*P* < 0.0001) as well as between the center of the palate and 4 mm away from the center (*P* < 0.001). The PBT decreased as we moved from the anterior palate (canine–first premolar) region to the posterior palate (first molar–second molar), as well as when we moved away from the midline. The only exception was between the canine and first premolar region where the PBT was significantly more 4 mm away from the center of the palate. Further, we showed that this pattern was uniform in all the experimental groups. Our findings differ from the findings observed by Gracco et al., who did not find a significant difference between the bone thickness at the suture, at 3 mm, and at 6 mm away from the suture in the different age groups [1]. Furthermore, we showed that we were not able to differentiate between growing males and non-growing males in the PBT at different points measured. Similarly, we were not able to differentiate between growing females and now growing females in the PBT. Kang et al. had results similar to our study, but observed the thickest bone away from the center, at the premolar region instead of the canine premolar region [25]. Further, they showed thicker bone in the middle of the palate compared to the paramedian region especially posterior to the premolar region [25]. Similar to our results, other studies have shown maximum bone thickness away from the center (3 to 4 mm away) at the canine and premolar region [26–28]. Comparison of the PBT between the males and females revealed that the males had significantly higher (*P* < 0.001) PBT than the females. The pattern of sexual dimorphism was consistent between the growing and non-growing males when compared with their respective counterparts of growing and non-growing females. Our findings were different from those of Gracco et al. and Ryu et al. where they found no gender-related differences in the bone thickness, and this could be attributed to different mean age of males and females in our study [1, 8]. Moreover, we were unable to differentiate the PBT between the growing and non-growing populations in both the sexes. One could conclude from this finding that very little bone is formed or added after age 13 (which was our mean age for the growing population).

Bone density measured by pixel intensity values (PIV) or CT numbers on CBCT scans is a practical method to assess density from CBCT exams. While PIVs are conceptually similar to Hounsfield units (HU) that are measured on CT scans, Hounsfield units are derived values that are calculated on images obtained from multi slice CT scans based on a complex mathematical post processing formula. Since such an option is not yet computable on CBCT volumes, its best to use pixel intensity values while keeping in mind that these are not absolutely defined like the Hounsfield units but never the less are practical because many studies have previously shown that Hounsfield units and pixel intensity values have a linear relationship. With careful evaluation and by using some internal controls to define the tissue thresholds, PIV values can be a very reliable way to assess bone density [29].

The PBD was significantly higher in between the canine and first premolar area in the center of the palate in all the four experimental groups. Similarly, the PBD was significantly higher in between the second premolar and first molar and the first molar and second molar at 4 mm away from the palate in all the experimental groups. The bone density was higher as there was more cortical bone present (Fig. 2a, b) in the canine and first premolar region (center of palate). We also found considerable variation in the PBD value within each group, like the previous studies; however, given our much larger sample size, it is safe to assume that our group means are more precise and represent the population [24]. Surprisingly, our finding shows that growing female and non-growing female had more PBD than growing male and non-growing male respectively. The bone matrix of the newly formed bone is less mineralized, and subsequent primary and secondary mineralization (increase in bone density) continues for years after the growth (increase in bone thickness). In our research, the mean age of growing females/non-growing females was more than the mean age of the growing males/non-growing males, and after bone formation, the mineral content rapidly increases up to 70% of full mineralization within 1 month of bone formation, but secondary mineralization which contributes to 30% of mineralization lasts for years and probably females being older in our sample size had more secondary mineralization [30, 31]. Moreover, approximately 8% of bone turnover (bone remodeling) occurs annually and bone turnover is activated at different time points in each individual [32]. Further, non-growers (male and female) had significantly more palatal bone density than their growing counterparts. Similarly, Han et al. showed significantly higher bone density in adults than in adolescents [24].

### Clinical significance

The PBT and PBD vary between males and females. The palatal bone thickness was higher in the anterior part of the palate (between the canine and first premolar), so there are chances that bone to mini-implant contact area will be more; thus, primary stability of the mini-implant will be more. However, at the same time, the palatal bone density is highest in between the canine and first premolar area, thus will require higher torque for the insertion of mini-implants. Our suggestion will be to place the mini-implants with increased diameter in the anterior part of the palate (good bone quality and quantity) to prevent fracture of the mini-implants.

## Conclusions

1. The anterior part of the palatal bone has the highest bone thickness (bone quantity) in all the four groups. Growing males had significantly higher bone thickness when compared to growing females between the canine and first premolar and between the first premolar and second premolar. Similarly, non-growing male has the higher bone thickness when compared to growing and non-growing female.
2. Non-growing males and non-growing females had a significantly higher bone density in the center of the palate at all the four sites of measurements, when compared to growing males and growing females respectively.
3. Bone density was significantly higher in between the second premolar and first molar and between the first molar and second molar in all the four experimental groups, 4 mm away from the palate.
